# The correlation between postural control and upper limb position sense in people with chronic ankle instability

**DOI:** 10.1186/s13047-015-0082-9

**Published:** 2015-06-18

**Authors:** Shmuel Springer, Uri Gottlieb, Uria Moran, Guy Verhovsky, Ran Yanovich

**Affiliations:** Department of Physical Therapy, Ariel University, Ariel, Israel; Israel Defense Force Medical Corps, Zerrifin, Israel; Institute of Military Physiology, IDF Medical Corps, Tel-Hashomer, Israel

**Keywords:** Proprioception, Ankle-sprain, Chronic ankle instability

## Abstract

**Background:**

Chronic ankle instability (CAI) is attributed to functional instability driven by insufficient proprioception. However, it is not clear whether the deficits are related to global impaired performance or to specific decrease in ankle motor-control. The aim of this study was to assess the correlation between lower limb postural control and upper limb position sense among people with CAI, in order to further explore the function of the central neural control in people with CAI.

**Methods:**

Fourteen participants (10 males, 4 females) with self-reported CAI and 14 age- and gender-matched, healthy controls participated in this study. Each participant completed single-limb stance postural control tests and shoulder position sense tests. The Overall Stability Index (OSI) was used as a measure of postural stability. The average of the absolute error score (AES) was calculated as a measure of shoulder position sense. Pearson correlations between the scores of the four body sites –lower limb postural stability (preferred/non-preferred), shoulder (preferred/non-preferred) were determined separately for each group.

**Results:**

In the control group, significant correlations were found between the OSI score of the right and left ankles (r = 0.887, *p* < 0.001), between the AES of the right and left shoulders (r = 0.656, *p* = 0.011), as well as between the OSI score and the AES of the non-preferred side (r = 0.649, *p* = 0.012). In the CAI group, significant correlation was found only between the OSI score at both ankles (r = 0.6, *p* = 0.002).

**Conclusions:**

Individuals with CAI demonstrated lower limb postural control and upper limb position sense similar to those shown in healthy controls. However, correlations between the lower and upper limbs were observed only in the healthy controls. Clinicians can use this information and employ activities that focus on coordinating the upper and lower extremities when designing neuromuscular control training programs for people with CAI.

## Background

Ankle sprains occur frequently in athletes and active-duty soldiers, as well as among the general population [1, 2]. Although often considered minor, the long term consequences of ankle sprain may have major impacts on health and daily life [3]. For example, 72 % of people post-ankle injury reported that they were functionally impaired by their ankle, in most cases unable to perform sports at a desired level [4].

Recurrent ankle sprains occur in 70 % of individuals that have experienced a lateral ankle sprain previously. The cause of this high level of recurrence is currently unknown [5]. Individuals who report on residual symptoms, which include repetitive episodes of ankle joint instability and feeling of ‘giving way’, have been termed as having chronic ankle instability (CAI) [6]. It has been suggested that this CAI can be attributed to functional instability driven by insufficiencies in proprioception and postural control [7], which can be defined as the inability to maintain stability above a narrow base of support in single-limb stance [8]. However, two recent systematic reviews of postural stability, including a meta-analysis, that aimed to determine whether postural control is adversely affected in those with CAI, indicated that such deficits have not been detected consistently in this population [9, 10]. Therefore, it was recommended that the clinical diagnosis of CAI should not be based solely on static postural control testing, but rather on more challenging and complex evaluations of sensorimotor performance [9].

Previous reports that tested proprioception in able-bodied participants, have presented conflicting results. Some studies suggested that proprioception is site-specific, meaning that there is likely to be a common control program for the same joint on the two sides of the body, and that the program uses proprioceptive information from sources that are specific to those joints [11, 12]. Contrary evidence, however, suggests that proprioception may be a general body attribute. Hence, it is expected that participants with proprioceptive deficits at one site may have generally poor proprioception at other body sites [13, 14]. Another finding related to proprioception is side-general asymmetry, in terms of non-dominant side proprioceptive superiority. This phenomenon has been demonstrated by several recent studies that evaluated lower and upper limb joints [12, 15, 16]. Studies of people with ankle injuries indicated bilateral associations of unilateral injury by demonstrating sensorimotor deficits of both injured and uninjured ankles [17, 18]. However, it is not clear whether the deficits in the uninjured ankle are related to global impaired performance, or to a specific decrease in motor control in the ankle joints. To the best of our knowledge, upper limb proprioceptive abilities and the connection between sensorimotor performance in the upper and lower limbs, among people with recurrent ankle injuries have not been evaluated previously.

The aim of this study was to assess the correlation between two aspects of sensorimotor function, lower limb postural control and upper limb position sense in participants with recurrent ankle sprains. This evaluation can contribute to a better understanding of sensorimotor function in this population, and may provide knowledge to effectively evaluate and treat recurrent ankle injuries.

## Methods

### Participants

The study included 28 participants, a group of 14 with CAI and 14 age- and gender-matched, able-bodied controls. The enrolment criteria for the CAI group were based on inclusion criteria for investigating CAI as suggested by Delahunt et al. [6]. Participants were recruited for the CAI group if they had: (i) a history of at least one significant ankle sprain which occurred at least 12 months prior to enrolment in the study and was diagnosed by a physician or a physical therapist using clinical examination classifications described by Malliaropoulos et al. [19], (ii) a history of at least two episodes of ‘giving way’, and feelings of ankle joint instability [6] in the previously injured ankle joint of 1 year post-initial sprain, (iii) the most recent injury occurred more than 6 weeks prior to the study enrolment, (iv) the ability to apply full weightbearing on the injured lower extremity with no more than mild discomfort. Exclusion criteria for this group were: evidence of a concomitant injury (such as a bony injury or significant muscular/tendon injury), previous ankle surgery, other pathological conditions or surgical procedures in either upper/lower extremity, neurological/vestibular or any other balance disorder. The control group included healthy participants with no current or previous conditions that could affect proprioception, in particular: the presence of joint injuries within the past 6 months, a chronic disease (e.g., multiple sclerosis, stroke, Parkinson’s disease, rheumatoid arthritis, or type 2 diabetes), or any history of visual or vestibular disturbance affecting balance.

Both groups included only participants who demonstrated right upper and lower limb preference. Handedness was measured using the ten-item version of the Edinburgh Handedness Inventory (EHI) [20]. Laterality score for participants was an EHI +50 to +100 (where scores of +100 represent an extreme right hander and scores of −100 represent an extreme left hander). Footedness was measured using the Waterloo Footedness Questionnaire (WFQ) [21], scores from +7 to +20 indicate right-footed (where scores of −7 to −20 indicate left-footed, and scores between −6 and + 6 indicate mixed-footed).

The study was approved by the Israel Defense Force Medical Corps Ethical Review Board (approval number IDF-1379-2014). All participants provided written informed consent to participate in the study.

### Assessments

Postural assessment was carried out using the Biodex Stability System (BSS) (Biodex Corp, NY, USA). The BSS is comprised of an unstable support platform that allows up to 20° of multi-axial surface deflection. The BSS can be set at 12 levels of stability, with 12 the most stable foot platform setting and 1 the least stable. The measures of postural stability were obtained at stability level 3 and included the overall stability index (OSI), which measures the variance of foot platform displacement in degrees in all directions: sagittal plane anterior/posterior and frontal plane medial/lateral stability. This stability index represents the mean angular displacement of the platform in degrees from the zero-point position. Higher scores indicate greater difficulty maintaining the platform in a stable position; hence, poorer balance stability. Conversely, lower scores represent better stability and better balance. The reliability of the BSS has been established, with an intraclass correlation coefficient ranging from 0.72 to 0.81 [22].

**Fig. 1:**
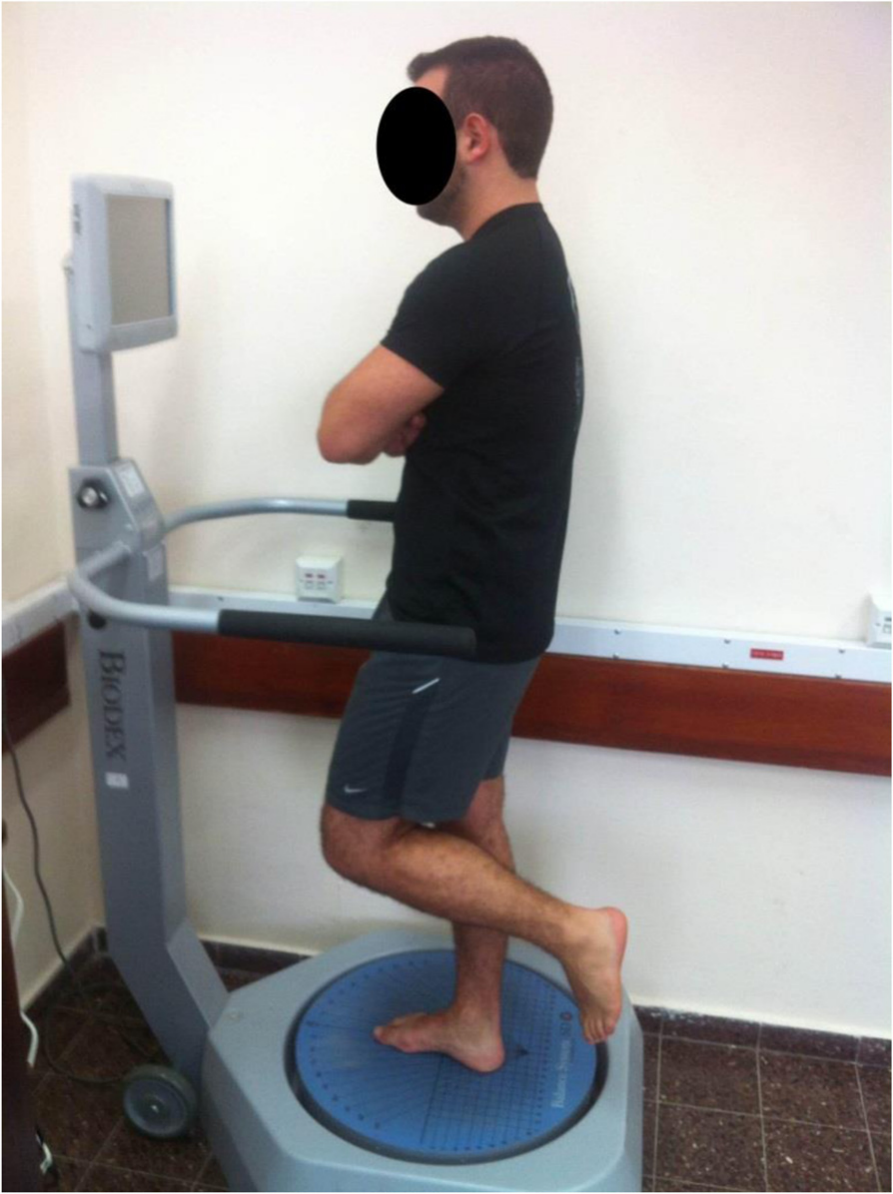
Postural control testing. Note. Participants stood in single-limb stance in the central region of the Biodex Stability System platform. Participants were instructed to look straight ahead and to keep the platform as motionless as possible for 20 sec

The postural control testing was performed with the participants barefoot, in single-limb stance, in the central region of the platform while keeping the unsupported limb in a comfortable position so as not to contact the tested limb or the test platform (Fig. 1). Participants were instructed to look straight ahead and to keep the platform as motionless as possible for 20 s. Each participant was given a familiarisation session. Two measurements were taken for each limb in random order, with a two minute rest interval between trials. The average of the two evaluations was used for data analysis. This method has previously been used to assess postural control in participants post ankle sprains [23, 24].

**Fig. 2:**
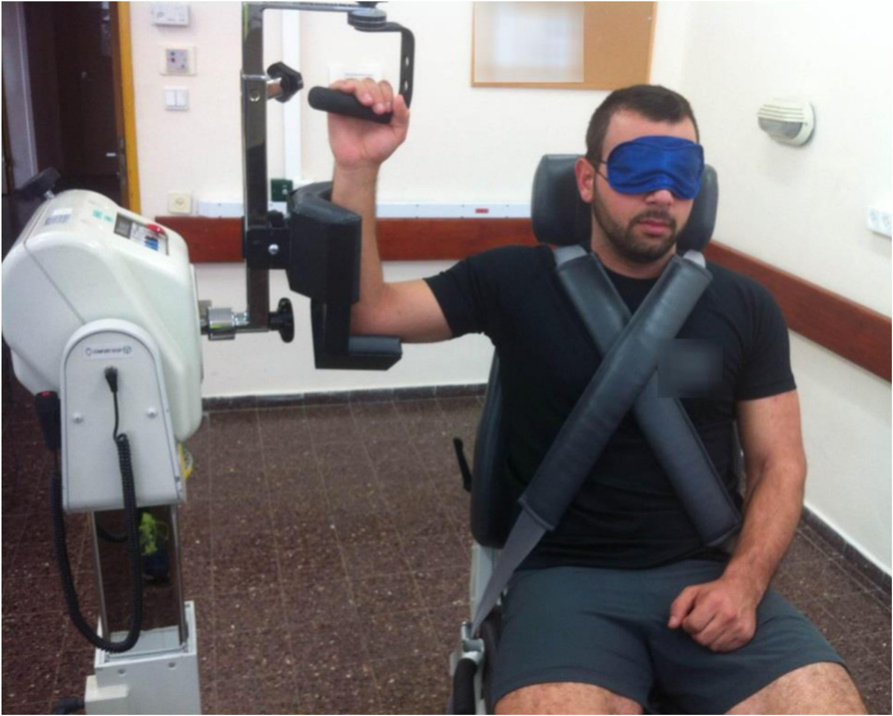
Shoulder position sense testing. Note. Participants were secured into the seat of the Biodex Multi-Joint System and then the shoulder was positioned at 90° of abduction and 90° of external rotation in the plane of the scapula, with the forearm perpendicular to the floor. Participants were asked to actively reproduce the passively set index angle

The shoulder was chosen for measuring of upper extremity joint position sense, since, similar to the ankle, motor control plays a significant role in ensuring the joint stability [25]. The Biodex Multi-Joint System (Biodex Corp, NY, USA) was used for the position sense test of the shoulder. The system includes an electro-goniometer, which is sensitive to 1° increments [26]. Active angle repositioning was measured with the participants in a seated position, with their back vertical, the shoulder positioned at 90° of abduction and 90° of external rotation in the plane of the scapula (30° in front of the frontal plane), and the forearm perpendicular to the floor (90° of flexion at the elbow) (Fig. 2). This position was selected to simulate the abducted, externally rotated position of the shoulder required in many activities. Participants were blindfolded to eliminate visual cues related to joint position.

After setting the starting position, the participant’s shoulder was passively moved to one of the test angles (45° or 60° of shoulder external rotation) by the examiner. Participants were asked to concentrate on the sensation of the presented angle for three seconds. Then, the participant’s shoulder was passively returned to the starting position by the examiner. Following a three second rest period, the participant was asked to actively reproduce the presented joint angle. Once the participant felt that the shoulder was in the position of the presented angle, he/she pressed the hold switch, preventing the dynamometer from further movement. Each subject underwent a short mock test (45° of shoulder external rotation) with each limb to ensure comfort and understanding of the test protocol. Then, the procedure was repeated twice more for the 45° target angle (three times in total) and three times for the 60° target angle. Measurements were taken with both limbs in a randomised order with a two minute rest between trials. The average of the absolute error score (AES) was calculated (i.e., the difference between the reproduced angle and the target angle) and used for data analysis. Previous research has found this technique to be an accurate and reliable method of measuring shoulder joint position sense [25, 27].

### Data analysis

The Anderson-Darling test was used to assess data normality; however due to the lack of normal distribution, nonparametric analysis was used. The Mann–Whitney test was applied to compare baseline characteristics as well as the performance (i.e., OSI and AES) between the CAI and control groups. The performance in the non-preferred/left (upper and lower limb measures) versus the preferred/right for each group was compared using Wilcoxon matched pairs test. Likewise, the Wilcoxon matched pairs test was used to test for differences between groups. This test was also employed to compare the OSI in the limb with recurrent sprains versus the unaffected limb, in the CAI group. Pearson correlations between the scores of the four body sites, namely lower limb (preferred/right and non-preferred/left) and shoulder (preferred/right and non-preferred/left), were determined for each group separately. Finally, the Z test was used to determine whether any difference existed between the correlations in the CAI group versus the control group. SAS V9.3 (SAS Institute, Cary, NC, USA) was used for statistical analyses. Significance was set at *p* < 0.05.

## Results

Data on participant characteristics are summarised in Table 1. There were no differences in baseline characteristics (age, BMI, EHI handedness score, and WFQ Footedness score) between the CAI and control groups.

**Table 1:** Participant characteristics (median and interquartile range) and comparisons

Characteristic	Group		P-value
	CAI (n = 14)	Control (n = 14)	
Age (years)	20.0 (1.0)	20.3 (1.8)	0.418
BMI	22.5 (3.2)	20.9 (2.8)	0.312
Gender (F/M)	4/10	4/10	---
Handedness	95.0 (27.5)	100.0 (17.5)	0.707
Footedness	11.0 (5.5)	14.5.(8.8)	0.122
Ankle with recurrent sprains (RT/LT/BIL)	6/6/2	---	---
Time (weeks) since last sprain	8.0 (3.5)	---	---

*CAI* chronic ankle instability, *RT* right, *LT* left, *BIL* bilateral

### Effect of group (CAI vs. Control), side (non-preferred/left vs. preferred/right), and affected limb

Table 2 presents the median and interquartile range of the measured outcomes of each group, as well as comparisons between groups. Comparisons of the performance in the non-preferred/left side vs. the preferred/right side for each group, and for the entire sample, are also provided. Comparisons (OSI right/left and AES right/left) demonstrated no difference in all outcomes between the CAI and the control group.

**Table 2:** Group outcome measures (median and interquartile range) and comparisons

Parameter	Group		Comparison - CAI vs. Control *P*-value	Comparison - preferred/right vs. non-preferred/left *P*-value		
	CAI (n = 14)	Control (n = 14)		CAI group (n = 14)	Control group (n = 14)	Entire sample (n = 28)
OSI RT	1.35 (0.63)	1.20 (0.78)	0.945	0.134	0.237	0.051
OSI LT	1.45 (0.55)	1.25 (0.73)	0.518			
AES RT	3.83 (2.03)	4.59 (1.65)	0.089	0.761	0.552	0.534
AES LT	3.92 (1.49)	4.68 (2.38)	0.223			

*CAI* chronic ankle instability, *OSI* overall stability index, *AES* absolute error score, *RT* right, *LT* left

Comparisons of the performance in non-preferred/left vs. the preferred/right side yielded no effect for side (i.e., right/left, see Table 2). Though no significant effect was found for preferred/non-preferred side, a strong trend was demonstrated for superiority of the right preferred side when analysing the results of the OSI in the entire sample (*p* = 0.051).

The comparison of the OSI in the limb with recurrent sprains versus the unaffected limb included the 12 participants in the CAI group who had unilateral recurrent sprains (see Table 1). No difference was found between the OSI score in the two limbs (*p* = 0.490).

### Correlations between body sites

Pearson correlations between the scores of the four body sites yielded differences between the CAI and control groups (Table 3). In the control group, significant correlations were found between the OSI score for the right and left foot (r = 0.887, *p* < 0.001), between the AES for the right and left shoulder (r = 0.656, *P* = 0.011), and between the OSI score and the AES for the non-preferred/left side (r = 0.649, *P* = 0.012). In the CAI group, a significant correlation was found only between the OSI score for the right and left foot (r = 0.600, *p* = 0.002). The Z tests, indicated that there was a significant difference in correlations between the two groups only in the correlation of the OSI score and the AES for the non-preferred/left side (*p* = 0.037).

**Table 3:** Pearson correlations between the mean scores of the lower limb (preferred/right and non-preferred/left) and shoulder (preferred/right and non-preferred/left)

a. CAI				
Parameter	OSI RT	OSI LT	AES RT	AES LT
OSI RT	1	0.600	0.040	−0.335
		*p* = 0.023	*p* = 0.893	*p* = 0.242
OSI LT		1	0.321	−0.114
			*p* = 0.264	*p* = 0.698
AES RT			1	0.480
				*p* = 0.083
AES LT				1
b. Control				
Parameter	OSI RT	OSI LT	AES RT	AES LT
OSI RT	1	0.887	0.262	0.420
		*p* < 0.001	*p* = 0.365	*p* = 0.135
OSI LT		1	0.448	0.649
			*p* = 0.108	*p* = 0.012
AES RT			1	0.656
				*p* = 0.011
AES LT				1

*CAI* chronic ankle instability, *RT* right, *LT* left, *OSI* overall stability index, *AES* absolute error score, *RT* right, *LT* left

## Discussion

This study investigated the sensorimotor function of different body sites in participants with recurrent ankle injuries. As noted in recent systematic reviews [9, 10], postural stability measures in single-leg stance did not discriminate between participants with CAI and those without, as well as between the limb with recurrent sprains versus the unaffected limb in the CAI group. It should be noted, however, that while the measure of static postural stability may not be sensitive enough to detect deficits associated with CAI, more dynamic assessments, such as the single-leg-hop stabilisation maneuver, may have the ability to defer between individuals with CAI and individuals with stable ankles [28, 29]. In addition, reports of postural assessment through the Balance Error Scoring System have also shown promise in detecting differences between those with and without CAI [30].

Similar to lower limb postural stability results, there was no difference in the shoulder position sense between the CAI and control groups. This is consistent with the findings of Hung et al. [31], who found that people with unstable shoulders can perceive active shoulder angles as accurately as those with healthy shoulders.

While the sensorimotor function of the different body sites was similar in both groups, examination of the correlations between the body sites differentiates the groups. In the able-bodied group, Pearson correlations showed significant positive correlations between the same joint on the right and left sides, as well as significant positive correlations between the upper and lower limb in the non-preferred/left side. This may suggests a site-specific and a non-preferred side attribute in the way the brain integrates proprioceptive information. However, in the CAI group there was no correlation between the upper and lower limb and significant positive correlations were found only between the lower limb on the right and left sides.

It has been suggested that joint injury may be more likely to occur when there is a “pre-existing, global deficit” in proprioception [14]. It is not clear whether individuals with CAI have a “pre-existing, global deficit”. However, the lack of correlation demonstrated in the CAI group may suggest difference in the sensorimotor integration and processing post-injury, when compared to healthy participants. Dynamic movements involving multiple body segments, such as locomotion, require controlling and coordinating the arms and legs to accomplish a rhythmic, smooth, movement pattern [32]. Indeed, it has been shown that people with recurrent ankle sprains may have a typical altered gait pattern that might be related to altered control of the central nervous system [33, 34]. Furthermore, in a study that compared the effect of dual tasking on postural performance in participants with CAI and a matched control group, concurrent performance of a cognitive task decreased postural stability only in the participants with CAI [35]. This may also suggest a deficit in central neural control for maintenance of balance in that group. To our knowledge, the present study is the first to describe the lack of correlation in sensorimotor function in a sample of participants with recurrent ankle sprain.

The greatest challenge presented by CAI may not be in treatment, but in prevention [36]. A recently published position statement by The National Athletic Trainers Association, intended to provide recommendations for conservative management and prevention of ankle sprains, indicated that clinicians should implement a multi-intervention injury-prevention program that focuses on balance and neuromuscular control to reduce the risk of ankle injury [37]. Our results suggest that this multivariate approach should include sensorimotor exercises and tasks that coordinate the upper and lower extremities. For example, throwing a ball toward a specific target, while standing in a single limb stance on a wobble board or soft surface. It is also recommended that accurate assessment and documentation of progress of such activities should be a standard part of ankle-rehabilitation programs.

It has been suggested that there is non-preferred limb superiority in the utilisation of proprioceptive feedback. The advantage of the non-preferred limb is attributed to the functional differences between the roles of limbs especially in bilateral tasks. The non-preferred limb usually stabilises a specific position to enable the preferred limb to manipulate and perform a task [16]. For example, while hammering a nail or kicking a ball. Thus, joints in non-preferred limbs are more likely to receive more “positioning” practice, resulting in more accurate discrimination of movement. The results in the control group, which demonstrated correlation between the upper and lower limb only in the non-preferred (left) side may support this ‘superiority’ hypothesis. Nevertheless, as reported by previous studies [25, 38], the study results did not demonstrate differences in the tested performances between the preferred and non-preferred shoulder and ankle. A possible explanation may be related to the joints tested and the evaluation method in the current study. Proprioceptive asymmetry was mainly evident at distal joints and under non-weightbearing conditions [39–41]. However, the present study included only one distal joint (i.e., the ankle), which was evaluated in a weightbearing condition. Furthermore, the evaluation was of postural control that is affected by proprioception as well as by the motor control system. Therefore, it is not surprising that asymmetry was not reported in the present study. Future studies with multiple joints should be conducted to evaluate whether proprioceptive asymmetry exits.

The present study has several limitations. Firstly, different aspects of sensorimotor function were evaluated in the upper (i.e., position sense) and lower limb (i.e., postural control). When testing sensorimotor function and acuity, it is important that the tests maximise external validity (i.e., the similarity between the laboratory and real life function) [42]. The shoulder test was selected to simulate the abducted, externally rotated position of the shoulder required in many sporting activities and the ankle test was chosen as it has the advantage of testing in the weightbearing position. The similarity of these tests to normal function enhances the external validity of the current study. Nevertheless, future investigations in people with CAI should examine inter-limb correlations using the same aspects of sensorimotor function. Secondly, the study cohort consisted of a relatively small sample, with a narrow age range, and it included only participants who demonstrated right upper and lower limb preference. Thirdly, while the enrolment criteria for the CAI group were based on self-reporting of ‘giving way’ and feelings of ankle joint instability, it did not include the use of an ankle instability questionnaire, such as the identification of functional ankle instability (IdFAI) [43]; By not using the ankle instability questionnaire eliminated our ability to quantify this aspect of perception. Future studies with larger and varied samples that also confirm self-reported ankle instability with a validated ankle instability-specific questionnaire, are warranted.

## Conclusions

Participants with CAI demonstrated lower limb postural control and upper limb position sense similar to those of healthy controls. However, correlation between lower and upper limbs was observed only in the healthy controls. These results may be explained by a deficit in the central neural control of sensorimotor integration and processing in people with CAI. Clinicians can use this information when designing neuromuscular control training programs for people with CAI and potentially reduce the risk of re-injury.

## Abbreviations

CAI: Chronic ankle instability
OSI: Overall stability index
AES: Absolute error score
EHI: Edinburgh Handedness Inventory
WFQ: Waterloo Footedness Questionnaire
BSS: Biodex Stability System
SD: Standard deviations
BMI: Body mass index
